# A deep reinforcement learning account of competition and integration of visual and goal vector signals for spatial navigation

**DOI:** 10.1038/s41598-026-63080-3

**Published:** 2026-07-27

**Authors:** Sandhiya Vijayabaskaran, Sen Cheng

**Affiliations:** Institute for Neural Computation, Faculty of Computer Science, Ruhr University Bochum, Bochum, Germany

**Keywords:** Engineering, Mathematics and computing, Neuroscience

## Abstract

Integrating different sources of information is essential to successful spatial navigation. For instance, animals often rely on a combination of visual impressions, self-motion, olfaction, and other signals to navigate towards a goal. This is especially important when navigating in uncertain environments, where switching from one source of information to another or integrating multiple sources of information may be required to make navigation decisions. We propose a deep reinforcement learning model of the interaction of visual and goal-vector signals based on reinforcement learning and use it to study behavior and spatial representations. We show that the nature and degree of signal noise strongly influences how the signals drive behavior and spatial representations. Our model also demonstrates that the ability to navigate using each information source independently, in addition to integrating them, is crucial to successfully navigating in uncertain environments. Counterintuitively, our model shows that when one of the signals is removed, navigation may be improved if the remaining signal is reliable and sufficient to navigate. However, this improvement comes at the expense of robustness. Our modeling results demonstrate that combining redundant sources of information in biological systems is far more complex than suggested by sensor fusion in the engineering context.

## Introduction

Animals make use of several available sources of information and take into account various sensory modalities to navigate: Visual, self-motion, odor, tactile, magnetosensory perception, and so on could all be used by animals to navigate their surroundings^1^. Intuitively, being able to use multiple sensory signals increases the chances of successfully navigating in uncertain environments. For instance, humans rely on a combination of visual, auditory, and proprioceptive cues to maintain balance and navigate through their surroundings^2^. The ability to dynamically rely on alternative sensory signals when one becomes unavailable can greatly improve navigation capabilities, especially in unpredictable environments. However, successfully integrating different information sources to form a coherent representation of the environment is far from straightforward. The reliability and accuracy of each source can vary, and animals must constantly evaluate and prioritize which information to rely on in any given situation, and also be able to deal with conflicting information^3^.

In neuroscience and behavioral research, this issue has been primarily studied in the context of how vision and self-motion information interact to influence navigation and spatial representations^4–7^. Behavioral studies show that when animals navigate using visual cues, they still manage to reach the goal when the lights are turned off at the start or during navigation^8,9^. Similar behavior is observed in humans, where participants can navigate blindfolded to a previously seen goal^10^, demonstrating that both vision and self-motion can be used to navigate to the goal.

While it is clear that vision and self-motion signals interact, the precise nature of this interaction remains unknown. One question is whether the two signals cooperate or compete—are both signals somehow integrated to determine which direction to move, or is one signal selected over the other based on some criteria^1^ ? In the case of the former, it is also unclear how and to what extent each of the signals contributes to the integration, and in the latter, which signal is chosen and how. Experimentally, both outcomes have been observed in behavioral studies in rodents: An integration of both signals, with visual cues dominating, as well as a selection of one of them^5,]^^11–]^^13^. Both kinds of interactions have also been observed in behavioral experiments in humans. Although some studies show that humans combine vision and self-motion to navigate^14,15^, others suggest that one or the other is preferred^16,17^. In general, the behavioral evidence is consistent with the integration of signals when the mismatch between them is small and selection when the mismatch is intermediate or large.

Neuroscientists, on the other hand, have focused on linking place cell firing to these information sources. Again, both visual cues and self-motion have been shown to influence place cell firing, although to different extents. On one hand, multiple studies have shown that place cells consistently fire at a fixed distance from the start location of a linear track, even when the position of the start box is shifted^18–20^. However, various visual cues, such as the position of a cue card, the introduction of barriers, and environmental borders, have also been shown to strongly modulate the location of place cell firing^21,22^.

The observed differences may be due to the difficulty of fully dissociating the two signals in experiments, but a few studies have explicitly addressed this question using lesions^23^, virtual reality^24^, and genetically modified animals^25^. Although these studies clearly demonstrate that both vision and self-motion jointly impact place cell firing, they reach different conclusions regarding the relative contributions of the two, which could potentially be attributed to the different types of interventions involved. This disparity in experimental findings is also reflected in computational models of place cells, which make different assumptions about the underlying mechanisms. While some models are based on the assumption that self-motion information drives place cell firing^26,27^, others propose that visual cues play a predominant role^28–30^.

Computationally, the integration of multiple signals has often been modeled from a Bayesian perspective^31,32^, in which movement or heading direction is estimated based on the system’s beliefs about the reliability of each signal. Under the Bayesian perspective, optimally integrating two signals results in a weighted combination of the two, with the more reliable signal being associated with a higher weight. This framework, however, requires making explicit assumptions about the priors.

In this study, we address some of these issues with a computational model based on deep reinforcement learning (RL). RL is a framework in which an agent learns to maximize rewards from its environment through trial and error^33^. When combined with deep neural networks, this approach gives rise to deep RL, which has also been used to generate insights for neuroscience^34^. Deep RL is particularly well-suited for studying spatial navigation, as both share several fundamental characteristics, i.e., they both involve goal-directed behavior, internal representations guide actions, and learning requires effective credit assignment, i.e., identifying which decisions in the past led to successfully reaching the goal. These properties have led to a growing number of recent studies that examine navigation through the lens of deep RL^35–]^^38^. We use this approach in the present study to model the interaction between visual and self-motion information in a navigation task. While we model this interaction for vision and self-motion in particular, the modeling approach and results can be extended to include other potential information sources available to the agent as well.

## Results

**Fig. 1:**
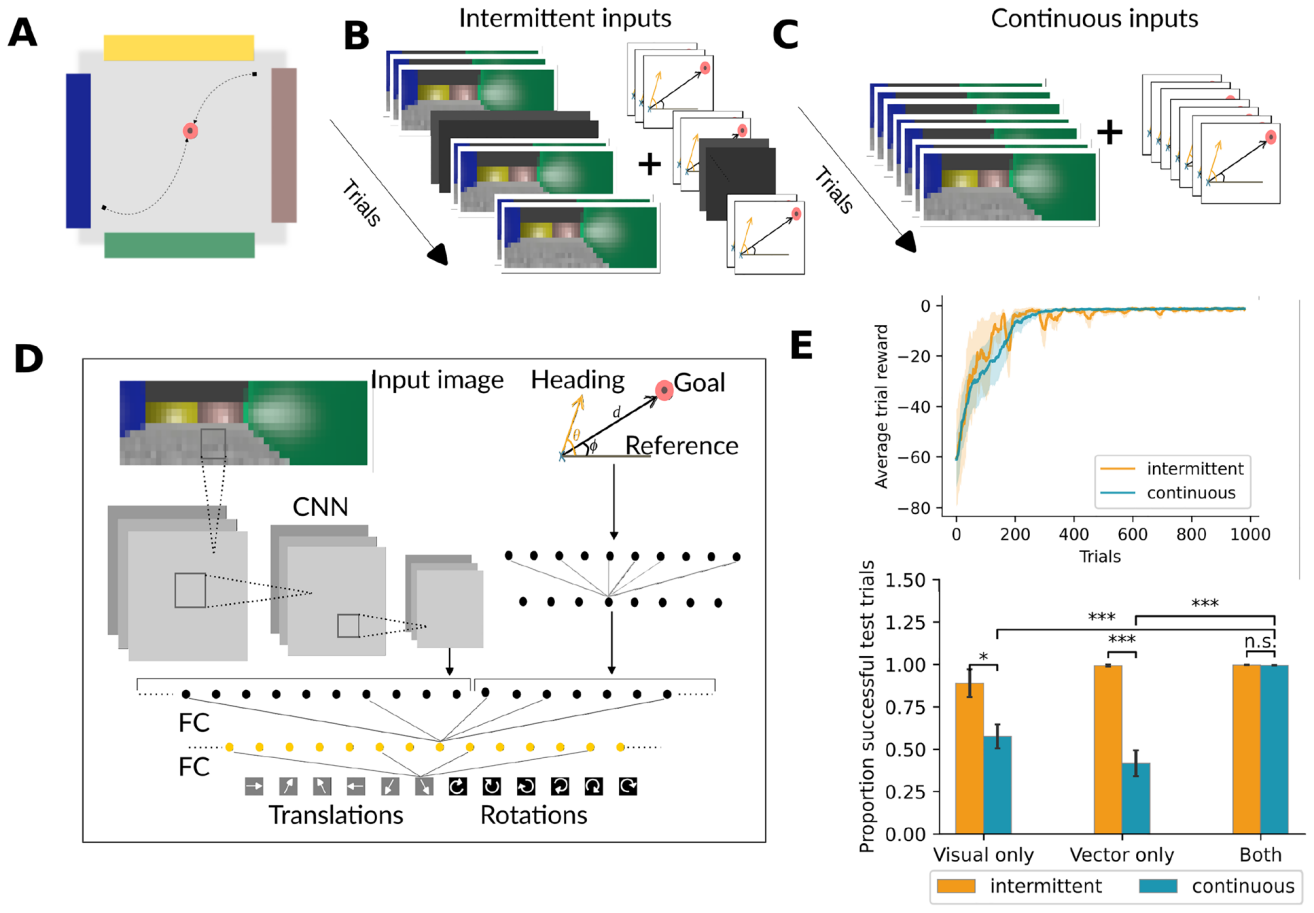
Task variants and computational model (**A**) The guidance task. The agent must navigate to a fixed, unmarked goal from different start locations in the environment. At every time step, the agent receives a visual and goal vector input from the environment to guide navigation. (**B**) Task with intermittent input signals. During training, either signal may be lost intermittently, effectively forcing the agent to learn to use both signals to navigate. To simulate the temporary loss of a signal, input to either the vector or the visual stream is randomly turned off for 10 trials every 30 trials. (**C**) Task with both input signals continuously present. (**D**) Model architecture. The network has two separate streams that process visual and vector inputs before integrating them. The visual stream uses a convolutional neural network (CNN), and the vector stream uses a fully connected network (FC). The two streams are then combined and jointly processed. The final layer consists of action units that determine the direction of translation (grey icons) or rotation (black icons) of the agent. Translation actions move the agent by a fixed distance in one of six directions, whereas rotation actions rotate the agent in-place to face one of these directions. For simplicity, only some selected connections are shown in all layers. (**E**) Learning curves (top) and test performance (bottom) when the agent is trained with intermittent and continuous input signals. Bars show the mean proportion of successful test trials (success is defined as arriving at the goal node). The agent learns both tasks effectively, but only learns to navigate near-perfectly with the individual signals when it was trained with intermittent signal loss. Learning curves show mean reward for the first 1000 trials for 10 simulation runs. Shaded areas show standard error of the mean (SEM). Bars show mean±SEM, for 10 simulation runs. *p*-values were computed using a two-sample t-test with Bonferroni correction. *** *p*<0.001, ** *p*<0.01, * *p*<0.05.

### Computational model and task setup

We modeled how signals used for making navigation decisions interact with one another and guide navigation using deep RL^39^. Our computational model relies on two inputs to navigate to an unmarked goal in the environment—naturalistic visual inputs from the simulation environment in the form of RGB images and a *goal vector* that is updated at each step. The goal vector is a 3-dimensional vector $(d,\phi , \theta )$, where *d* is the distance to the goal, $\phi$ is the angle between the goal vector and a reference vector, and $\theta$ is the allocentric heading direction of the agent with the same reference vector. The goal vector is related to self-motion signals discussed in the Introduction as follows. Self-motion signals are used for path integration, which involves the continuous updating of an agent’s position and orientation by integrating the self-motion cues. On the other hand, a goal vector is used in vector movement, i.e., using a vector that indicates the distance and direction to the goal is to navigate^1,38^. Path integration and vector movement are intricately linked, since path integration is required to update progress toward the goal in vector movement, as well as identify when the displacement required to arrive at the goal has been achieved. However, it is important to note that despite their overlap vector movement and path integration are different processes. Path integration serves as a general purpose mechanism for spatial updating that can also occur outside the context of vector movement, such as localization within a metric map. Furthermore, vector movement includes an additional computation analogous to vector subtraction where the current position vector (potentially estimated via path integration) is subtracted from a start-to-goal vector. The latter may be derived from diverse sources, including long-term memory, path integration along a prior outbound trajectory, or, in the case of humans, verbal instructions. We simplify this picture in our model by directly supplying the RL agent with the goal vector, although we show that this goal vector can also be successfully learned through supervised training (Supplementary Fig. S1).

The agent receives feedback from the environment in the form of a scalar reward that enables it to learn optimal behavior through trial-and-error. Our reward structure is deliberately straightforward—a constant step penalty of −1 and a positive reward +1 for reaching the goal. We intentionally avoid reward shaping to prevent biasing the agent toward any particular solution. We use a navigation task which we refer to as guidance^1,38^, where the agent must navigate to an unmarked goal from different start locations (Fig. 1A), much like the Morris Water Maze^40^, a classical behavioral experiment used to assess spatial learning and navigation in rodents. In the water maze, rodents are placed in a pool of opaque water and must navigate to a hidden platform submerged just beneath the surface using spatial cues. Rodents find the sensation of being in water unpleasant and are thus motivated to find rather direct paths to the submerged platform. Similarly, our agent receives a constant negative reward for every time step, motivating it to find direct paths to the goal. We do not set out to model the specifics of the water maze in detail, but the broad goals of the task, i.e. find an unmarked goal using the shortest path, are similar.

The crucial element in our model is a feedforward network that processes the two separate input streams, combines them, and makes suggestions for the next action to take (Fig. 1D). We also use agents that only have a single input (either vector or visual) for establishing a baseline, but otherwise use the same architecture as the model shown in Fig. 1D. The final layer of the network consists of action selection neurons, which enable the agent to take translational and rotational actions to navigate in the environment. The translation actions move the agent by a fixed distance in one of six directions, whereas the rotation actions rotate the agent in-place to face one of these directions. We chose this action space because previous work demonstrated that it leads to better navigation performance and generalization in the guidance task^41^. After the agent has learned to navigate to the goal, we examine its behavior and the spatial representations that emerge in the network in order to understand how the agent uses the two input streams to make navigation decisions.

The agent was trained to solve the guidance task under two conditions—one where either the vector or visual signal may be intermittently lost, such that the agent must learn to use both signals to navigate optimally (Fig. 1B) and one where both signals are continuously present, such that either signal alone or a combination thereof could be used for navigation (Fig. 1C). The first setup with intermittent signals is designed to simulate environmental uncertainty. Humans and animals have to deal with navigating in uncertain environments, where one or more sensory signals may become unreliable or completely unavailable. This loss can be due to several factors, such as poor lighting conditions, occlusion of the field of view by objects in the environment, a lack of external cues that allow for the correction of the path integration signal, or even sensory impairment. In this scenario, in order to successfully navigate to the goal on all trials, including those where one signal is lost, the agent has to learn to navigate using each signal independently as well as using them together when both are present. The agent experiences a loss of signal one-third of the training time. We chose this intermittency schedule for practical reasons to allow the agent to learn using signals independently and together. We only expect minimal sensitivity to specific intermittency schedules as long as enough experiences with signals on and off are collected, since the DQN agent is trained via experience replay rather than online learning. We tested this sensitivity for different schedules (Supplementary Fig. S2). The task setup with continuously available signals, on the other hand, represents the other end of the spectrum and is reminiscent of most experimental set-ups, where, for instance, vision alone may be fully sufficient to navigate to the goal.

We found that the reinforcement learning agent is able to efficiently learn the task under both conditions as illustrated by the learning curves(Fig. 1E, top). Furthermore, after learning, we assessed the agent’s ability to navigate using either one of the input streams, or both together, in a test phase. Clear differences emerged in the two tasks. In the case of intermittent signals (Fig. 1E, bottom, orange bars), the agent navigates to the goal from multiple start locations using either visual or vector streams individually as well as both together. However, when trained with both signals continuously present (Fig. 1E, bottom, blue bars) the agent is only partially successful using the individual signals, performing better than an agent with random weights, but worse than when both signals are available (two-sample t-test with applied Bonferroni correction $p<.001$ for visual only vs. both and vector only vs. both). Additionally, in contrast to the agent with intermittent signals, it relies slightly more on visual input than vector input, although this effect was not significant (two-sample t-test with applied Bonferroni correction $p=.26$). All results were averaged across 10 different simulation runs with different random seeds.

**Fig. 2:**
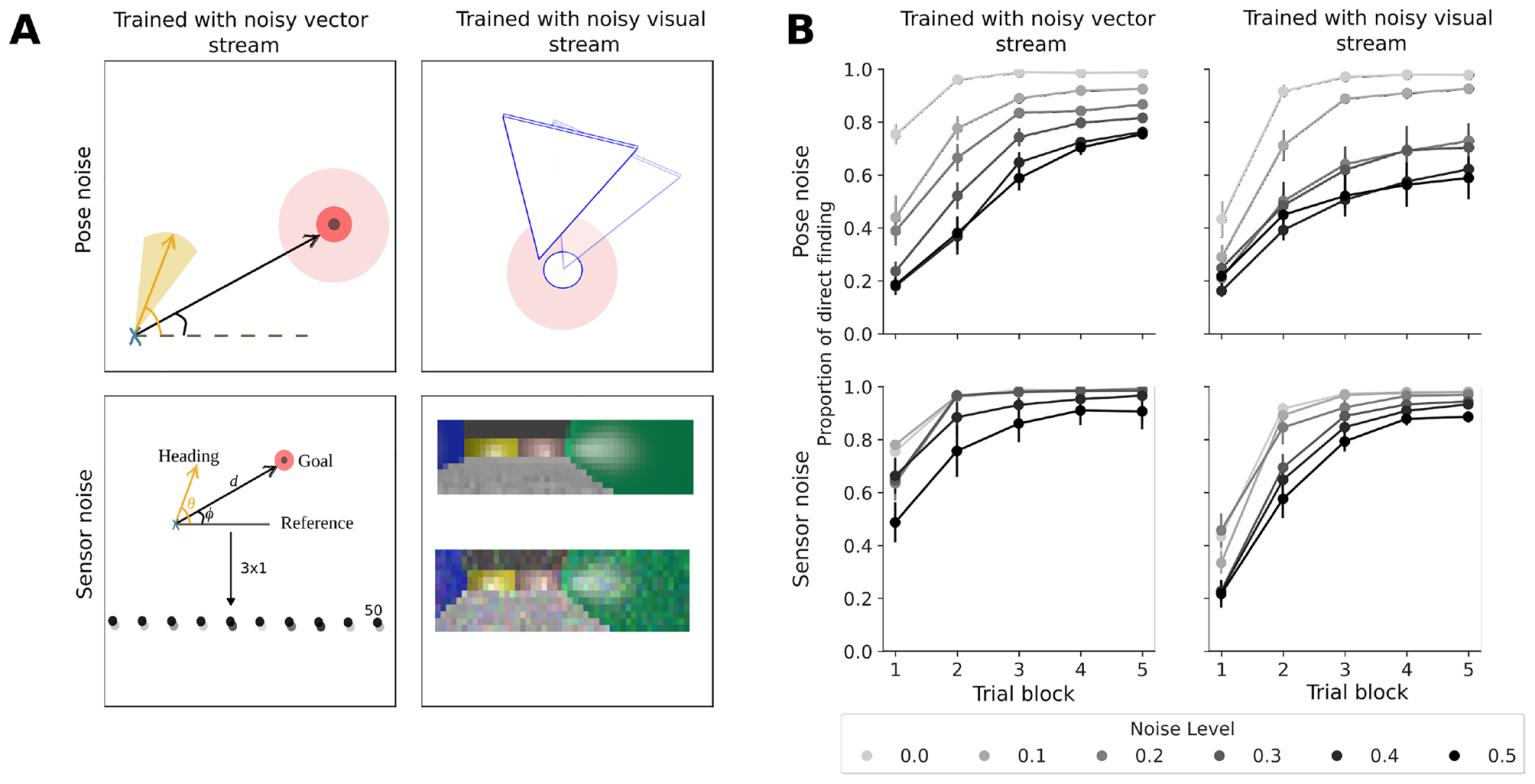
Adding pose and sensor noise to the inputs (**A**) Schematic illustration of the effect of the different noise types on the single streams. (**B**) Effect of pose and sensor noise on learning in agents with single input. Markers show the mean± SEM of direct finding performance in trial blocks consisting of 200 trials each. Darker lines indicate higher amounts of noise. In general, increasing the amount of noise in either vector input (left) or visual input (right) reduces direct finding performance during learning. Curves corresponding to zero noise are different on the left and right because of the networks accepting only vector or visual input respectively.

### Signal noise affects learning and behavior

We next investigated how noise in each of these signals affects the behavior of the agent. In order to establish a baseline, we trained agents that have only a single input stream, either vector or visual, to which increasing levels of noise are added. We added noise to each signal in two different ways, which we refer to as pose noise and sensor noise, respectively (Fig. 2A). Pose noise added to the vector stream corresponds to variability in the position of the goal as pointed to by the goal vector, while pose noise added to the visual input corresponds to variability in the camera position and bearing which captures the image presented to the agent at each position. We achieve this by adding noise to the components of the goal vector and by presenting a camera image sampled around the noisy current position of the agent, respectively. On the other hand, sensor noise is not directly tied to the position of the agent or the goal. For sensor noise in the vector stream, we add noise to the first layer of the network, which represents the initial input. For the visual stream, we add noise to the image pixels to simulate random variations in the visual field. One can think of pose and sensor noise as affecting the accuracy and precision of the input signal, respectively.

We evaluated the learning progress of the agent in the first 1000 learning trials. In every learning trial, the agent starts at a random position in the environment and attempts to navigate to the goal location. A trial ends when the agent successfully reaches the goal, or after 100 time steps, whichever comes first. We divided these first 1000 learning trials into 5 blocks of 200 trials each, and calculated the proportion of direct finding trials in each trial block. A direct finding trial was counted when the agent took the least number of actions from start to goal on that trial, plus a $30\%$ tolerance to account for random exploratory actions. From the direct finding performance, we can see that in general, learning becomes slower with increasing signal noise (Fig. 2B), as expected. Comparing the mean slope between the first and second trial blocks as well as the total area under the curve (AUC) for each noise level shows a significant effect of noise on learning (One-way ANOVA across noise levels; Slope: *F* = 3.067, *p* = .0105; AUC: *F* = 18.8254, *p* < .001). Furthermore, learning is slower in the presence of pose noise than with sensor noise for both vector input (two-sample t-test; Slope: $M_1 = 0.2560$ vs. $M_2 = 0.2650$, *t* = − 0.3208, *p* = .749; AUC: $M_1 = 3.1939$ vs. $M_2 = 4.4242$, *t* = − 9.4384, *p* < .001) and visual input (Slope: $M_1 = 0.2843$ vs. $M_2 = 0.4393$, *t* = − 5.5723, *p* = < .001; AUC: $M_1 = 2.7721$ vs. $M_2 = 3.7796$, *t* = − 6.8059, *p* < .001). Pose noise also lowers the asymptotic performance in general as indicated by the difference in the final block performance between noise types (two-sample t-test; $M_1 = 0.8072$ vs. $M_2 = 0.9592$, *t* = 0.8072, *p* < .001). Finally, we find qualitative differences in the influence of the noise type on vector and visual inputs. Pose noise has a greater impact on the asymptotic performance of agents trained with visual input than on agents trained with vector input (two-sample t-test; $M_1 = 0.8255$ vs. $M_2 = 0.7148$, *t* = 3.6313, *p* < .001). By contrast, sensor noise has no significant impact on the asymptotic performance between input types (two-sample t-test; $M_1 = 0.9666$ vs. $M_2 = 0.9422$, *t* = 1.5481, *p* = .125). We examine how these differences affect learning and signal integration in agents that simultaneously receive both inputs in the next section.

### Noise type and the availability of inputs influences how input signals are combined

We next ask how pose or sensor noise influence how agents with both input streams uses and combines the input streams when the input signals are intermittent. There are two sources of uncertainty to the agent in the intermittent case: uncertainty caused by the removal of either signal and uncertainty caused by noise added to either stream. These two manipulations are intended to capture distinct aspects: while signal intermittency reflects complete signal loss, forcing the agent to navigate with the remaining signal alone, added noise reflects measurement uncertainty in pose estimation and sensor readings. As an example, intermittency might correspond to whether a species evolved to navigate in varying conditions (e.g. daylight and darkness), whereas noise might correspond to sensor limitations (e.g. poor vision). We focus our analyses in the rest of this study to the latter, i.e., the effects of pose and sensor noise.

**Fig. 3:**
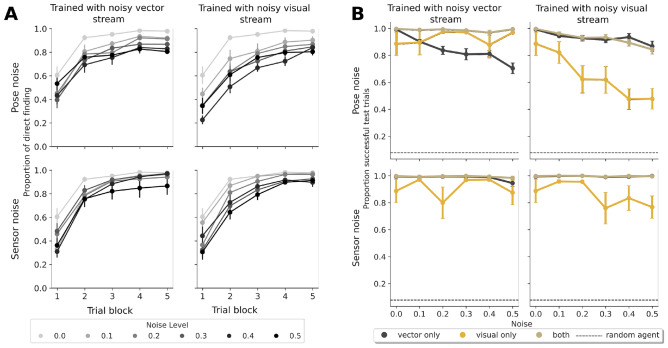
Learning and test performance when signals are intermittent. (**A**)Direct finding in networks trained with varying levels of input noise in the vector (left) and visual (right) input streams as a function of training trial blocks. Markers show mean±SEM. (**B**)Test performance with individual and combined inputs. Agents perform well above an agent with random weights (dashed line) in all the cases. Markers show mean±SEM.

The direct finding performance as learning progresses, Fig. 3A, shows a pattern similar to that observed in the learning curves in Fig. 2B, i.e., performance is generally lower for higher levels of noise (One-way ANOVA across noise levels; AUC: *F* = 8.9964, *p* < .001). We next examine the ability of the agents to navigate using the individual signals as well as both together in a test phase, where the navigation ability of the agent is tested with one input ablated. Note that in order to ensure fair comparison, all signals are noise-free in the test phase. Since the agent is forced to learn to use both signals independently as well as together when the signals are intermittent, we expect that the agent also learns to use the noisy signal to a certain extent in order to successfully complete the task. The test performance of the agents reveals that this is indeed the case, with the agent performing well above an agent with random weights (dashed black line) in all conditions (Fig. 3B).

In addition, in agreement with the results from the agents with single input streams, we find that pose noise has a greater impact on navigation using the noisy signal when compared to sensor noise (Fig. 3A) (two-sample t-test; $M_1 = 0.8689$ vs. $M_2 = 0.9415$, *t* = − 5.8007, *p* < .001). The proportion of successful test trials using only vector input drops with increasing pose noise in the vector stream as measured by the correlation between noise and successful test trials (Spearman’s r; *r* = − 0.5389, *p* < .001) , and likewise when pose noise is added to the visual input (Spearman’s r; *r* = − 0.5422, *p* < .001) (Fig. 3B, top). On the other hand, moderate levels of sensor noise in the visual stream actually have an overall positive effect on the performance. Note that in the absence of noise, although the agent learns to use both signals independently quite well, it still does slightly better with vector inputs than visual inputs (Fig. 1E orange bars and Fig. 3B, noise=0), although this effect is not significant. Moderate amounts of sensor noise in either stream counteract this to a degree (mean performance at zero noise = 0.8870 vs. =0.9550 at noise=0.2, however, this effect was not significant; two-sample t-test; *p*=0.44). (Fig. 3B, bottom). This pattern suggests that sensor noise has an effect that is consistent with regularization, i.e., preventing over-reliance on one signal and promoting the development of more general representations in the network.

Next, we examined how the two signals are combined when both inputs are continuously available to the agent. In this case, the agent does not necessarily need to learn to use both streams or integrate them in order to navigate successfully; either stream by itself is sufficient. A surprising observation is that although the goal vector is sufficient to guide the agent to the goal and is a far more compact and direct representation of the goal position, the agent still relies on both signals in order to navigate. We speculate that the network makes use of both streams because both are predictive of the goal location, and the optimization procedure lands on a combination that most effectively minimizes the loss. Since the visual stream has more parameters because of the need to process more complex input, and there are no architectural constraints such as a gating mechanism pushing the agent to rely on the simpler goal vector input, the learned solution naturally integrates information from both inputs.

**Fig. 4:**
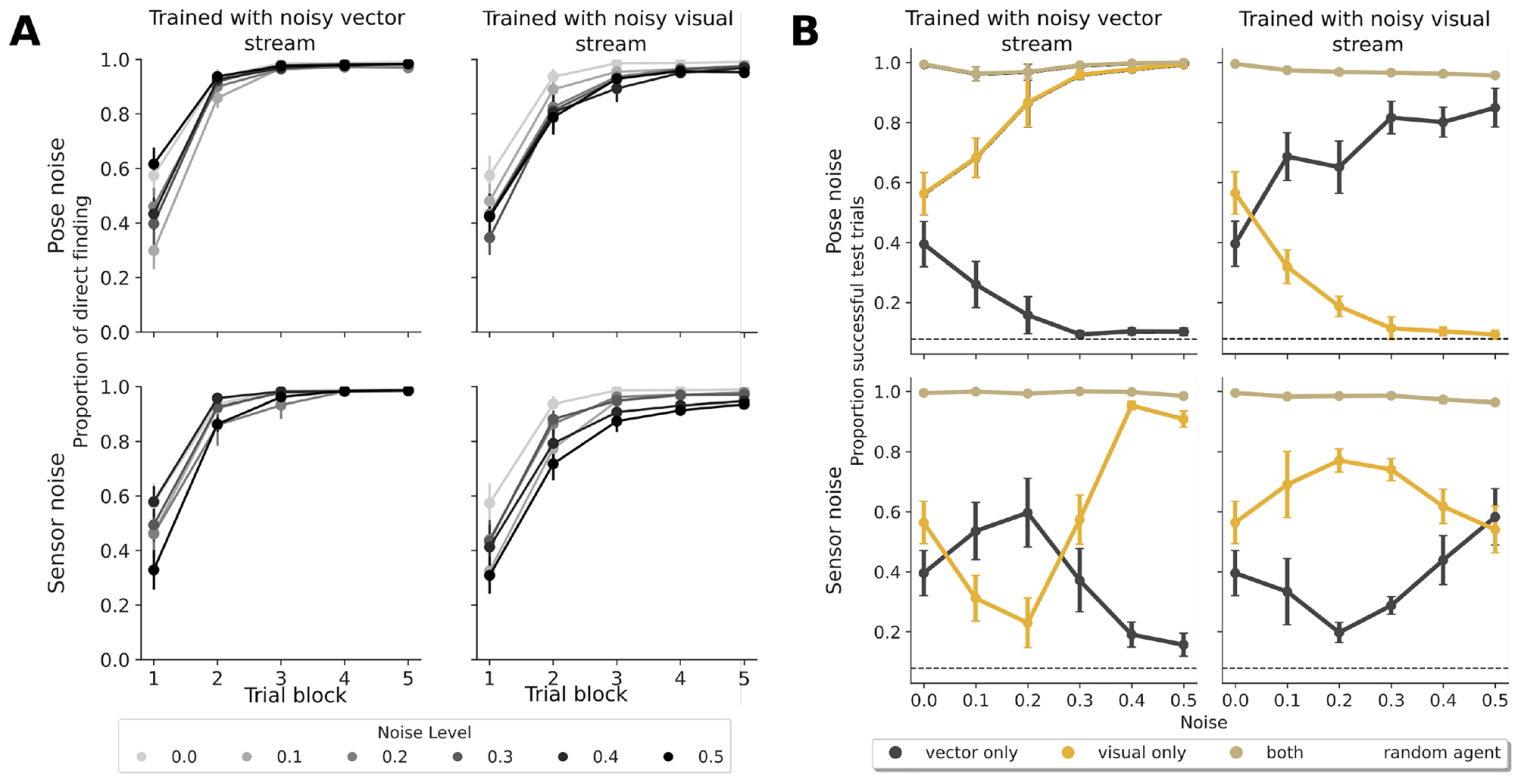
Learning and test performance when signals are continuously available. (**A**)Direct finding as a function of training trial blocks in networks trained with varying levels of input noise in the vector (left) and visual (right) input streams. Unlike when the inputs are intermittent, adding noise to one of the streams does not have a strong effect on direct finding, especially towards the end of learning. Markers show mean±SEM. (**B**):Test performance with individual inputs and combined inputs. Adding pose noise (top panels) leads the agent to rely more strongly on the noiseless signal, with navigation using the noisy signal driven down to the level of an agent with random weights for higher levels of pose noise. By contrast, sensor noise has a weaker effect on the noisy stream, but it paradoxically affects the non-noisy stream more than pose noise. Markers show mean±SEM.

There are also differences in the direct finding performance of the agent during training compared to the case with intermittent inputs—noise affects the performance to a much lesser degree (Fig. 4A) compared to when the signals are intermittent, as evidenced by the lack of correlation between the final block performance and the noise types (*p* = .744). The reason for this is, presumably, that the agent can deal with the noisier signal simply by relying less on it or ignoring it altogether. We examine if this is indeed the case in the test phase. As predicted by the previous results, pose noise strongly affects performance using the noisy signal (Spearman’s r; vector: *r* = − 0.5266, *p* < .001; visual: *r* = − 0.6773, *p* < .001), but with a key difference; the agent reduces its reliance on the noisy signal much more drastically, with the performance reducing to that of an agent with random weights for higher levels of noise (Fig. 4B, top). The test performance with sensor noise paints an even richer picture. While moderate amounts of sensor noise (evaluated at noise = 0.2) improve performance using the noisy signal (two-sample t-test; vector noise: $M_1 = 0.3947$ vs. $M_2 = 0.5960$, *t* = − 1.5355, *p* = .138 ; visual noise: $M_1 = 0.5633$ vs. $M_2 = 0.7700$, *t* = − 2.2356, *p* = .035), this improvement comes at the cost of performance when using the noiseless signal by itself (two-sample t-test; vector noise: $M_1 = 0.5633$ vs. $M_2 = 0.2290$, *t* = 3.0441, *p* = .0057 ; visual noise: $M_1 = 0.3947$ vs. $M_2 = 0.1970$, *t* = 2.0402, *p* = .052). This is apparent in the inverse-U-shaped and U-shaped test performance curves for the noisy and noiseless signals in the bottom panels of Fig. 4B. This pattern seen for sensor noise is again consistent with regularization.

In summary, studying the effect of noise on the two signals reveals a complex interaction between the two signals dictated by all three factors we consider: (i) Whether the signals are continuously or intermittently present during learning (ii) How the noise affects the reliability of the signal, i.e. position vs. sensor noise (iii) The nature of the signal itself, i.e., goal vector or visual input. Since there is no explicit gating or choice mechanism in the network, the extent to which each signal is used by the network emerges as a consequence of these factors, albeit constrained by the RL algorithm and architecture.

### Integration of sensory signals explains counterintuitive experimental findings in mice and rats

**Fig. 5:**
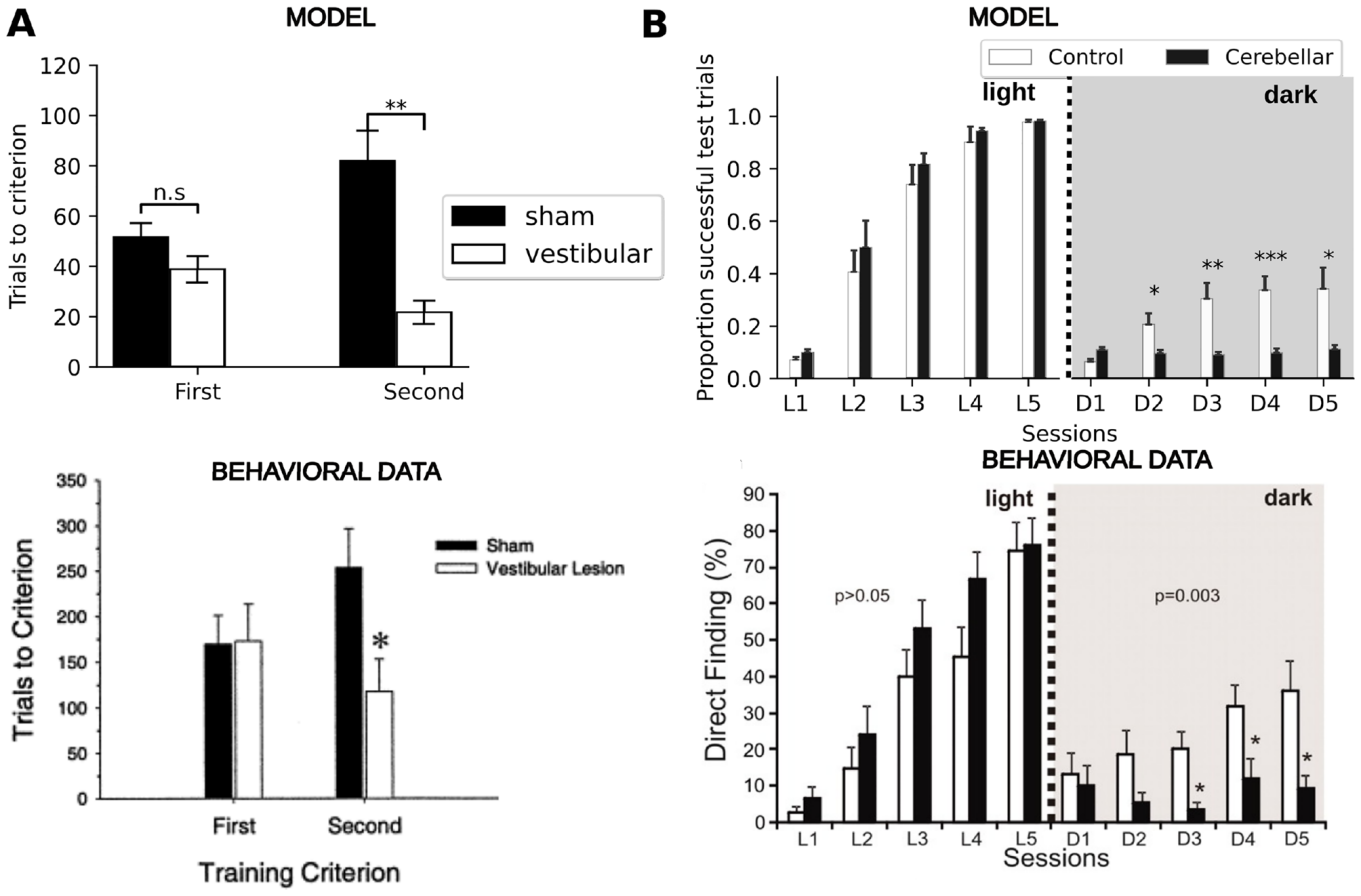
Discarding a noisy vector signal can explain counterintuitive findings from animal experiments. Modeling results are shown at the top, behavioral data at the bottom. (**A**) Rats with vestibular lesions found a hidden platform faster than controls did when the holding box was moved between trials^42^. Shown are the number of trials to criterion, i.e., lower is better. We used an agent with low vector noise to model the controls and one with the vector input removed for the vestibular lesions. Experimental results: Reproduced from Stackman & Herbert^42^) (doi: doi:10.1002/hipo.1112) with permission from Elsevier. (**B**) Mice with genetically impaired cerebellar function learn to find the goal faster than controls under light conditions, but fail to learn in dark conditions^25^. Shown are fraction of direct finding of the goal, so higher is better. An agent with low vector noise (signals are integrated) is used to model controls, while an agent with high vector noise (vector signal is largely ignored) is used to model the cerebellar mice. Experimental results: Reproduced from Rochefort et al.^25^, Science, 334(6054), 385–389. doi: doi:10.1126/science.1207403, AAAS. Statistical tests in the model were carried out using a repeated-measures ANOVA and Bonferroni corrected. ***p<0.001, **p<0.01, *p<0.05.

Our results indicate that in many cases, it is preferable to use a single stream to navigate when it is sufficient, rather than attempting to combine it with noisy redundant stream. We can use this finding to model two experimental findings in animals with impaired path integration. First, we model results from an experimental study that selectively disrupted the vestibular system of rats, thus presumably interfering with path integration^42^. The study found that the number of trials needed to meet a performance criterion (80% correct trials in two consecutive 16-trial blocks) was the same for both sham and lesioned rats when they were trained to find the goal in a Morris Water Maze starting from the same holding box position. However, there was a significant difference when the holding box position was changed between trials, with the vestibular lesion rats surprisingly doing better (Fig. 5A, bottom). We use the same performance criterion to evaluate our model.

We used the regular two-stream network with moderate vector pose noise (variance = 0.1) to model those with sham lesions, and a network with the vector stream removed, i.e., with only the visual stream, to simulate the rats with vestibular lesions. After the agent has learned to navigate to the goal from a single start location, like the rats starting from a single holding box position, we tasked the agent with finding the goal from four different starting positions. Our modeling results are qualitatively consistent with the animal experiments (Fig. 5A), with the vestibular-lesion model performing better in the second condition. First, there is only a small difference in performance between the agents when starting from a single start location, because (i) the task is relatively easy to learn, and (ii) a moderately noisy vector signal will still point in roughly the correct direction, which allows the agent to integrate the two signals and correctly navigate from the fixed start position to the goal. Thus, the pattern of results would remain qualitatively unchanged if the sham-lesioned model had a higher amount of added pose noise—up to the point where the agent starts ignoring the vector signal as shown in Fig. 4B (top left). After this point, the difference between sham- and vestibular-lesioned models would likely reduce drastically. Second, there is a large difference in the subsequent phase with four starting positions, animals with vestibular lesions and the agent with only the visual stream can adapt their behavior quite quickly and build upon the prior knowledge acquired during the first condition to navigate to the goal from different start locations. However, for the sham animals and agents with moderate noise in the vector stream, they must now learn how to integrate the noisy vector information from very different start positions, and the previously learned strategy for integrating the vector signal from a single starting location may actually interfere with learning a general strategy for correctly integrating this signal from any start position, thus affecting performance and slowing down learning.

Next, we simulated results from a different experiment in genetically modified mice that had impaired plasticity in the cerebellum. In the animal experiments, the genetically modified and control mice were tested on their ability to navigate to the goal in a Morris Water Maze under light and dark conditions^25^. We model the genetically modified mice with an impaired cerebellum with an agents with very high noise in the vector stream (variance = 0.8). In contrast to the network we use for modeling the experiment from Stackman & Herbert^42^, the impaired model has both streams present to allow it to still navigate in the dark condition. Similar to the previous simulations, we use an agent with moderate vector pose noise (variance = 0.1) as the control condition since path integration is inherently noisy. As in the animal experiment, we test both versions of the agent at different stages of learning, either with both visual and vector signals (light condition) or with only the vector signal (dark condition). When tested under light conditions, both agents improve their direct finding performance over time, similar to mice in the experiment. Our model also provides a hypothesis for the small but consistent advantage of the genetically modified mice in the light condition. This effect arises because agents with high noise in the vector stream learn to ignore the noisy signal quite early on during learning where the visual input is reliable, while models with moderate noise still integrate both signals and take longer to learn this integration. This slight advantage, however, comes at the cost of robustness, as evidenced by the poor performance of the model with high vector noise when tested in the dark, a problem observed also in genetically modified mice.

Thus, our model provides a mechanistic hypothesis about how impairments in the navigation system could counterintuitively lead to improvements in other cognitive areas. As with any computational model formulated at a relatively abstract level, it is designed to capture behavioral effects at a coarse grain, rather than to explain finer biological details, such as the mechanisms by which other brain areas might compensate for deficits, or the sources of individual variability in these responses. Additionally, since our model is trained from scratch, without any prior knowledge, it remains agnostic about the time course over which compensation for a noisy signal develops; whether on evolutionary, developmental, or shorter task-level timescales, as well as how interactions across these timescales might unfold.

### Spatial representations are differently affected by the removal of sensory input

We next set out to understand the types of spatial representations that emerge in the agents’ networks to support navigation and how the representations are modulated by visual and vector inputs. Place cell activity in rats is minimally disrupted in the dark^43^, and in congenitally blind individuals^44^. On the other hand, place cell activity is more dramatically disrupted by removing vector input^25,23^.

**Fig. 6:**
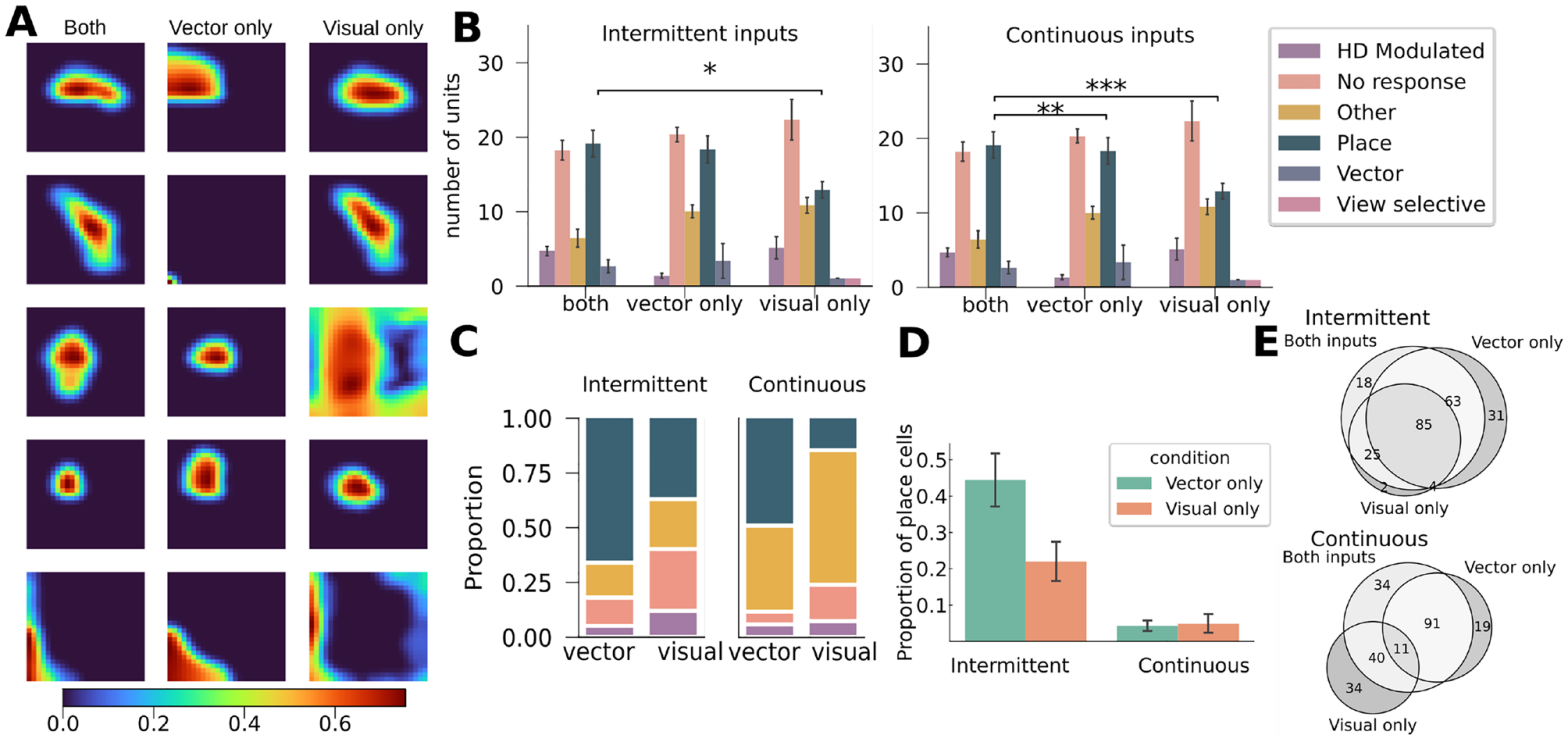
Place cell representations have a complex dependence on visual and vector inputs. (**A**) Different examples of place-like units that require both or either input to maintain their firing fields. Maps are smoothed with a Gaussian filter. Raw maps prior to smoothing and averaging over head directions are shown in Supplementary Fig. S3. (**B**)Distribution of response types in a network with both inputs, and with only one of the inputs. The counts were averaged across 10 runs. Removing either visual or vector input disrupts place cell representations. On average, this effect is more pronounced when vector inputs are removed (visual only). Markers show mean±SEM. *p-*values were computed using a two-sample t-test. ***p<0.001, **p<0.01, *p<0.05. (**C**)Transformation of place cells after removal of an input. The majority of place cells continue to remain place-like. The place cells that change to other response types typically either completely lose their response (silent), indicating that their firing was primarily driven by one input type, or the firing field loses its locality (“other”). (**D**) Proportion of place cells that maintain their place field at the same location after removal of one input. Almost all place cells remap in the case where the agent was trained with continuously available inputs. In the case where the inputs were intermittent, only partial remapping occurs, with more place cells maintaining their field location with vector input only. (**E**) Venn diagram showing number of place cell like units when network was trained with intermittent and continuous signals and tested using only visual input, only vector input, and both.

After training the agent on both intermittent and continuously present signals, we first computed spatial activity maps for all the neurons in the layer prior to the action selection layer and classified them (see "Methods"). A number of place-like units emerged in the network (Fig. 6) in both conditions, in agreement with previous work showing that an agent learning to navigate to an unmarked goal using visual landmarks tends to develop place-cell-like representations to support navigation^41^.

Removing either input results in some disruption of place-like units in the network; some of these units require both inputs to maintain their firing field, while others are able to maintain their firing field in the presence of one input alone (Fig. 6A). Removing the vector input results in a larger drop in the number of place cells than removing visual inputs (Fig. 6B) with both continuous and intermittent inputs, qualitatively consistent with experimental reports that place field firing is largely maintained in the dark^43^, but disrupted when the vestibular system is disturbed^23^. The degree to which place cells require one or both inputs to maintain localized firing however differs between agents which receive continuous and intermittent inputs, with a larger overlap in the case where both inputs are continuous. This is consistent with the agents needing to maintain separate representations in the case where inputs are intermittent, but developing more integrated representations when signals are continuously present.

We also examined what happens to place-like units after the removal of either input (Fig. 6C, E). In both cases, more units remain place-like with the vector input alone as compared to visual input alone. As suggested by Fig 6B the remainder of the units mostly either lose their local firing properties (“other”) or become silent. In addition, a small proportion of cells become head-direction modulated upon the removal of either input. That is, these units continue to have local firing fields, but the location of the firing field is now modulated by the heading direction.

Since the majority of the place-like units remain place-like even after the removal of one input (Fig. 6C), we next ask if those units maintain the location of their firing field or if they remap to new locations. We quantify the proportion of place cells that maintain the location of their firing field after the removal of input. When trained with intermittent inputs, many place cells continue to maintain the same firing field location with vector input only (Fig. 6D), much like place cells in rats navigating in the dark. Fewer cells show the same property with visual input only. Interestingly, the degree of overlap between place-cell-like units driven by different input signals differs between the intermittent and continuous input conditions (Fig. 6E). In the intermittent case, more place cells are “multimodal”, meaning that their spatial tuning is supported by both inputs. In contrast, under continuous input, substantially fewer place cells show such overlap, indicating a stronger segregation of input-specific representations that are then combined to produce the output. Removing one of the inputs in the continuous case thus leads to a disruption of the corresponding place cell population and consequently affects the downstream network output. In both the intermittent and continuous case, there are more units that are specific to vector input only, consequently, the removal of this input has a greater impact on the place cell population. We also computed the average similarity between the activation vector when both inputs are present and when one input is removed, and find a pattern consistent with the analysis of single units (Supplementary Fig. S4). Using linear centered kernel alignment^45^ to compute representational similarity shows that the vector-only representation is more similar to the both-input representation than the visual-only representation is. In the continuous case, representational similarity between the vector-only and visual-only conditions is low, as expected. The agent does not learn to represent spatial locations from either input independently, instead forming an integrated representation. In the intermittent case, however, similarity between the two single-input conditions is higher, consistent with the agent having learned to navigate using each input independently. Finally, representational similarity between agents trained on the intermittent and continuous tasks is high when both inputs are present, indicating that both training variants lead to comparable representations of spatial location overall. Taken together, these results suggest that in our model place field firing is modulated by both visual and vector inputs and that, overall, the removal of vector input results in a greater disruption of firing field locality and more remapping of place-like units.

**Fig. 7:**
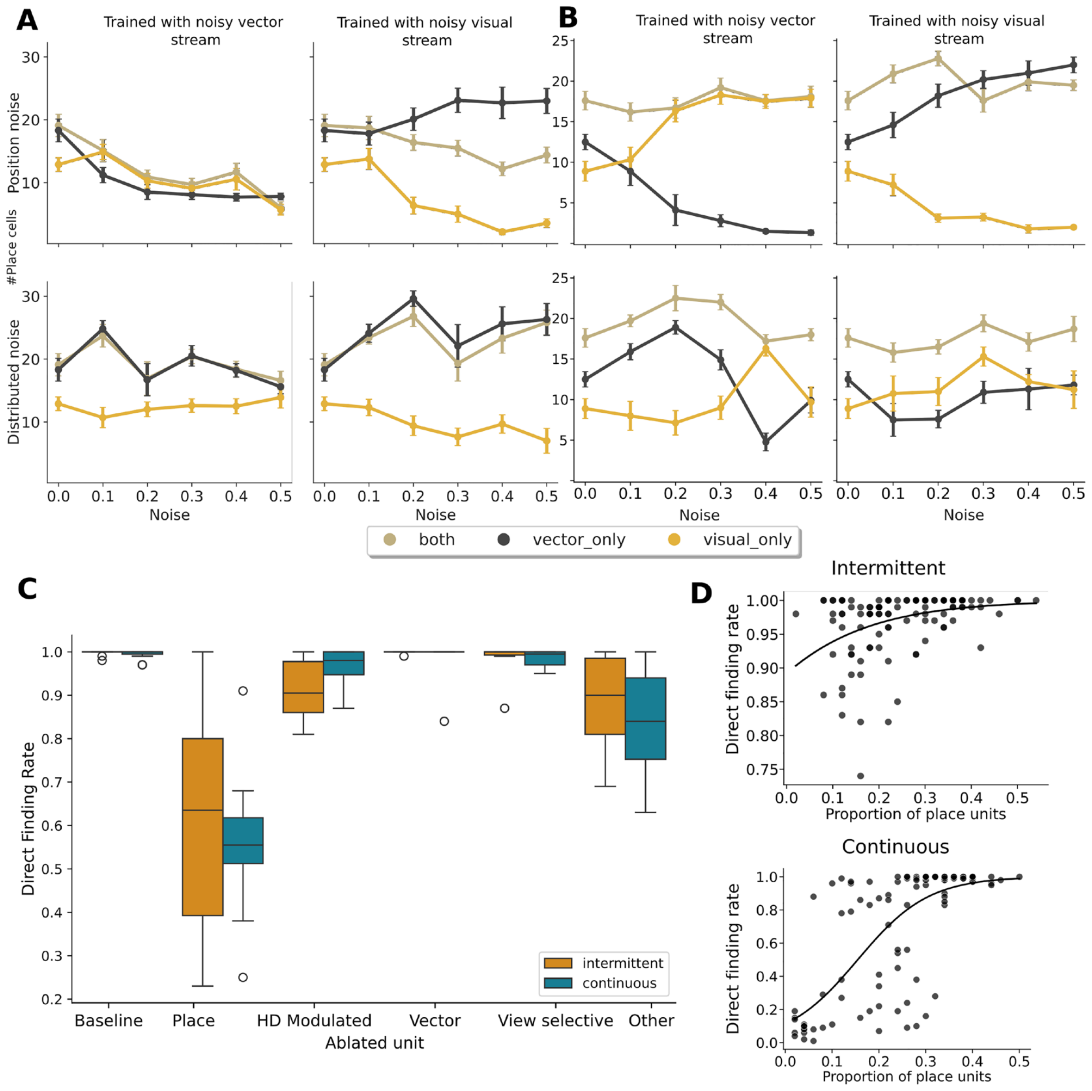
The functional role of place-like units in spatial navigation. (**A**) The effect of training noise on the number of place-like units, when input signals are intermittent. (**B**) Trained with continuously available input signals. The influence of noise on the number of place fields is determined by the signal to which the noise is added during training, and closely mirrors the behavioral performance (Figs. 3B and 4B). Markers show mean±SEM. (**C**) Results of ablating different unit types in the network. Removing place-like units has the largest effect on the performance of the agents in both the intermittent and continuous cases. (**B**) Correlation between direct finding performance and proportion of place-like-units in the network for the intermittent and continuous tasks. Results were fitted using binomial regression. Intermittent: coeff=6.2378, *p*<.001; Continuous: coeff=13.2477, *p*<.001.

Finally, we also looked at the effect of pose and sensor noise on the number of place-like units in the network (Fig. 7), which revealed that the number of place-like units closely mirrors the behavioral performance (Figs. 3B and 4B). This is an indication that the place-like units play a critical role in the navigation performance of the agent. We further test this hypothesis by perturbing the populations of units corresponding to each unit type, controlling for the number of units (Fig. 7C). This reveals that perturbing place-like units has the largest effect on the direct finding performance of the agent, in line with previous findings^41^. Correlating the direct finding performance with the number of place cells in the network also shows that the presence of place cells has a positive effect on the direct finding performance of the agent (Fig. 7D).

### Integration of conflicting sensory information

Agents in the physical world not only have to deal with noise in the inputs, but also with systematic deviations or biases. In this last set of simulations, we therefore study situations where the signals are systematically rotated with respect to each other, more specifically, the vector signal is rotated away from the goal, and thus, away from the direction indicated by the visual input. The agent was trained in the intermittent setting without any added noise for the conflict simulations. The vector input is rotated by rotating the angle of the goal vector, i.e., by changing $(d,\phi , \theta )$ to $(d,\phi \pm rot, \theta )$. The visual input remains unchanged, thus introducing conflict between the two signals.

**Fig. 8:**
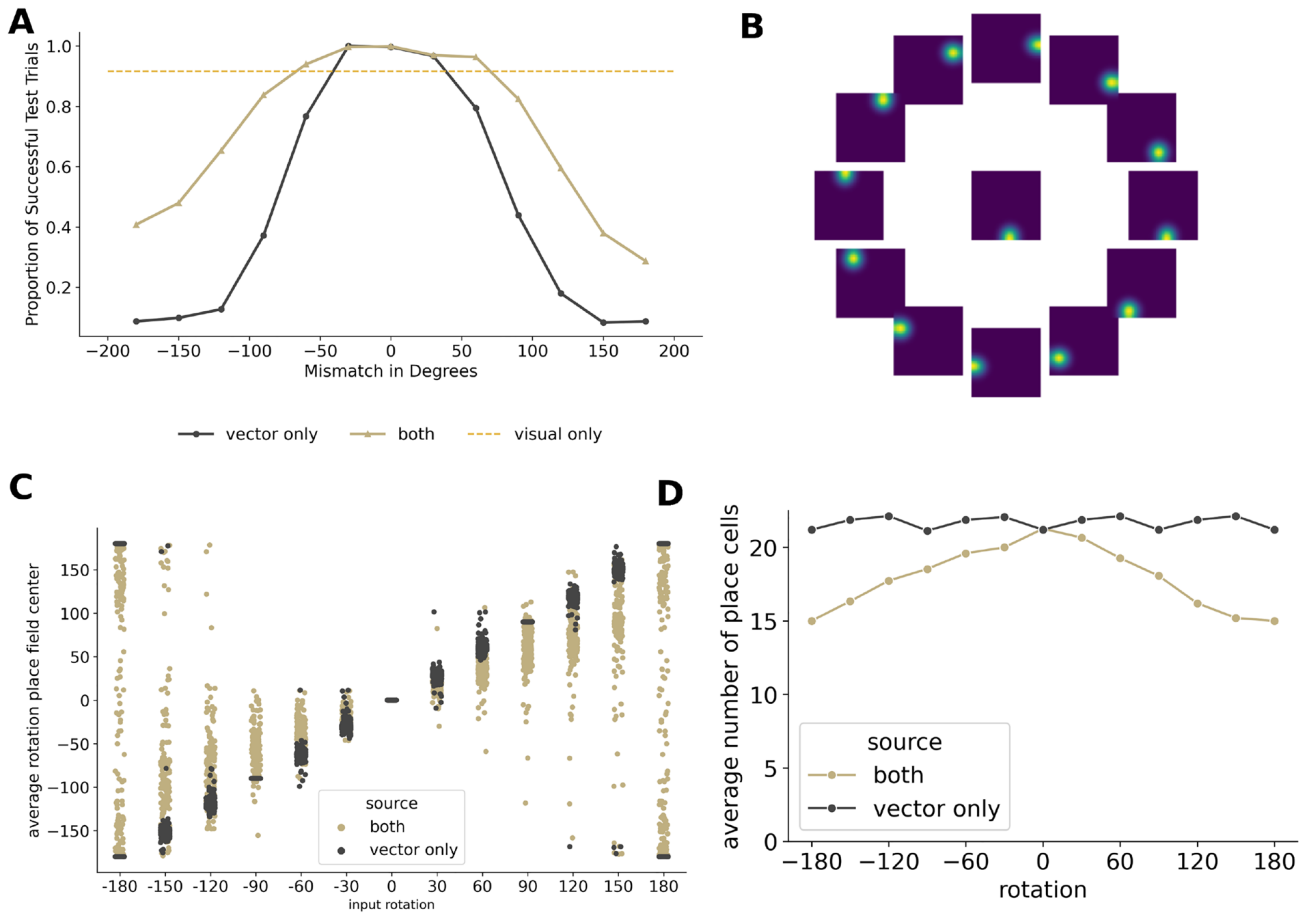
Effect of rotating the vector input (**A**) Test performance when inputs are rotated relative to each other. Each trajectory shows the agent performance with different amounts of rotation. The performance drops rapidly with angle of rotation while using the rotated (vector) input by itself, but this effect is mitigated while combining it with the stable (visual) input. (**B**) Example unit where fields rotate in tandem with the input. The center shows the unrotated field, while the surrounding fields correspond to a rotation by the amount indicated by its position relative to the center. Rotations were calculated with respect to the goal position. (**C**) Rotation of place field center with input. (**D**) Average number of place cells in the network with input rotation. The number of place cells drops with the angle of rotation while using both inputs but remains relatively stable using only the rotated input.

As expected, the agent’s performance using the rotated vector input rapidly deteriorates as the angle of rotation increases, pointing further away from the goal (Fig. 8A, black line). However, by combining this with the visual input, which indicates the correct location of the goal, the agent is able to improve its goal finding performance above chance (Fig. 8A, brown line). Examining the underlying spatial representations reveals a potential mechanism for this behavior—place field centers rotate almost exactly by the amount of rotation of the input when the vector input alone is supplied (Fig. 8B, C). By contrast, in the presence of both inputs, many place field centers rotate by an amount much lower than the rotation of the input, with some place cell units even maintaining their firing field at the same location throughout. The rotation of the place field center was calculated as the angle between two vectors: one from the goal location to the place field center with the unrotated input, and one with the rotated input. We next examine how place cell counts change as a function of the rotation angle (Fig. 8D). This analysis reveals that number of place cells remains stable with the rotated i.e. vector input alone, in agreement with the place fields rotating and thus leading to faulty navigation. On the other hand, using both units results in a drop in the number of place cells as the angle of rotation increases, suggesting a more complex remapping including partial rotation as indicated by Fig. 8C as well as loss of firing fields.

In summary, the agent shows a surprising degree of tolerance towards conflicting sensory inputs, even though the agent was never trained on conflicting situations. We hypothesize that this capacity arises from the robustness of the learned representations, which in turn results from the agent being trained on intermittent input signals.

## Discussion

We have presented a computational model of the interaction of goal vector and visual information for navigation based on deep reinforcement learning. We examined the effect of sensor and pose noise on navigation performance and spatial representations. We found that, in general, the agent is more sensitive to pose noise than sensor noise and that whether the signals are integrated or one signal is chosen over the other is primarily modulated by the type of noise in the signals. Integrating signals results in more robust behavior, which is useful while navigating under uncertainty, but can sometimes prove to be disadvantageous in other situations when the discrepancies between the two streams are too large. In the latter case, it might be better to ignore one input altogether.

### Factors affecting the combination of information sources

Our simulations reveal that navigating using multiple sources is a complex and multifaceted problem, with environmental uncertainty, the type and amount of noise in each signal, and the nature of the signal itself playing a role. Here, we discuss in more detail each of these factors and their impact on navigation behavior. Environmental uncertainty was incorporated into our simulations by intermittently removing either the visual or goal vector input, and we find that the agent is able to cope well with these dynamic changes. In behavioral experiments, humans and animals have also shown the ability to adapt well to the removal of sensory inputs. Gerbils navigating to food locations using an array of landmarks still reached their goal when the light was cut off mid-trajectory^8^. Similarly, rats trained to solve the Morris Water Maze were able to navigate accurately in the dark after starting their trajectory in the light^9^, and blindfolded humans can navigate to a target that they had seen prior to being blindfolded^10^.

In contrast to manipulating vision, stopping path integration and the estimation of a goal vector can be quite challenging. One alternative is to temporarily lesion structures necessary for path integration. For instance, Stackman et al.^23^ find that rats with lesions to their vestibular apparatus show the ability to navigate accurately to a goal when visual cues are present, but fail to do so in the absence of these cues, while those with sham lesions have no difficulty doing so. This technique, however, impairs path integration and, thus, the goal vector update for the entire duration of navigation, not mid-navigation like our simulations. Even so, it is likely a reasonable modeling assumption that the goal vector will not always be available during navigation. This is due to the fact that accumulating noise can easily degrade the path integration signal, which necessitates periodic resets by environmental boundaries or recognizable landmarks^46,47^. Thus, environmental uncertainty is an important factor that influences how signals interact to inform navigation decisions.

We find that the amount of noise in each information source also affects how signals are combined. In the model, depending on how much noise is present, a signal is either integrated with the other (more reliable) signal or ignored. This observation is closely related to studies that aim to identify whether signals cooperate or compete in order to determine movement direction. This is typically accomplished by bringing visual and vector (path-integration) signals into conflict with one another. Multiple studies have shown that signals are integrated for low and moderate mismatches and compete for higher mismatches^5,12,14,16,17^, which is qualitatively consistent with our finding that for lower noise levels, the two signals are integrated (cooperation), but at higher noise levels one signal is selected over the other (competition).

Finally, our model demonstrates that in cases of competition, it is not only the mismatch that determines which signal is selected but also the nature of the noise. Pose noise is much more detrimental for spatial navigation than sensor noise. It is quite possible that different types of noise are more likely to affect different sensory modalities. For example, visual information could be affected by sensory noise quite easily by adding noise to units in the processing stages of the visual system, but would be quite difficult to add pose noise, which would require shifting the visual representation as if the location were at a different location. On the other hand, such a shift might be much more easily introduced into the system representing vector information. Such differences in sensitive to noise types might account for some of the observed differences in how much animals rely on various modalities. In one study, visual information was preferentially selected over vector information in animal experiments^48^. Another study has shown that in rats, there is a hierarchical organization of visual, odor, and vector information^49^. Taken together, our results provide an account of the integration of signals that is neither purely cooperation nor competition but rather a hybrid of the two mediated by the factors discussed above.

### What drives spatial representations?

While the primary focus of this study was on behavior, our findings can also add to the current understanding of what drives spatial representations in the brain. Place cells and other spatially selective cells do not exist in a vacuum, as previous research has shown; rather, the nature of the task at hand and the navigation strategy used both interact to shape spatial representations^41^. Thus, place cells do not necessarily imply the existence of a cognitive map, rather, they emerge to support the requirements of the task at hand. Our model further extends this idea by demonstrating that sensory inputs and their primacy and reliability can also influence spatial representations. This is in line with several studies showing that path integration signals^18–20,24,]^, taste^50^, odor^51–55^, auditory^56^, haptic^57^, and visual cues^21,22,24^ all affect hippocampal place cell maps. Specifically, with regard to vision and self-motion, modeling and experimental work have shown that vision alone is sufficient to drive place representations in a smaller proportion of cells^24,41^. Our current study suggests that the dependence of place-like spatial representations on each signal is heterogeneous, with a greater reliance on vector input to maintain the firing field. This is qualitatively in agreement with Chen et al.^24^’s study in virtual reality, which found that overall, vision alone was sufficient for 25% of CA1 place cells, with the remaining 75% requiring input from self-motion, although to varying extents.

This larger reliance of place cell firing on the vector input in our model may reflect differences in how the vector and visual signals are formulated: the vector provides a compact and explicit representation of the goal’s relative location, whereas similar relevant spatial information must first be extracted from vision before it can be used for navigation. An interesting question is if a similar asymmetry may also to some extent explain the differences in how place cells are driven in biological species: self-motion signals are used for updating spatial variables, i.e. position and orientation, while extracting equivalent information from visual input remains more complex and may involve additional processing steps. To what extent this difference in signal structure accounts for the observed differences remains an interesting open question that could be addressed by future empirical and computational work.

Different species will rely on various sensory inputs to varying degrees, so we might anticipate variations in the kinds of spatial representations and behavioral performance based on the primacy and reliability of various sensory signals. Indeed, in species with a highly developed visual system, such as primates, hippocampal cells respond less often to a particular location and more commonly to a particular view^58^. On the other hand, in insects, the selection and integration of cues seems to emerge altogether without the need for such representations, and can to a large extent be explained by ring-attractor style models^59–61^.

Finally, further complicating the issue, many hippocampal cells exhibit mixed selectivity for a variety of additional, non-spatial, task-related variables, including lap number in a multi-lap run^62^, route taken^63,64^, position of another animal^65,66^, accumulated evidence for a choice^67^, and frequency of sound^68^. Thus, teasing apart exactly what factors drive spatial representations and to what extent will require further modeling and experimental work. In summary, while place cells underlie the selection and integration of signals in our model, in biology, this very likely depends on the species, task demands and the types of sensory inputs.

### Limitations and relationship to other models of multisensory integration

How to best combine and use information from multiple sensors in order to guide decisions is a question that has also been widely explored in engineering, notably in robotics. This problem, known as sensor fusion, is still an area of active research and development, with much effort being focused on improving the accuracy and reliability of navigation in mobile robots^69^. While these models are typically concerned with combining estimates from multiple sensors that measure signals from the environment, e.g. sound and vision^70^, our set up combines vision with an internally computed variable, i.e. the goal vector. Nevertheless, the general principle of how to best combine these estimates is a common goal of both approaches. Commonly used methods include Kalman filters and Bayesian approaches^32,70–72^, among others, and are often inspired by sensory integration in nature^73^. One of the most straightforward settings to model multisensory integration is linear cue combination, where signals are combined as a reliability-weighted average in a Bayes-optimal fashion^72,74^.

Bayesian frameworks have also been used to model multisensory integration in humans and animals^75^ and, in general, provide an account in which signals are combined by an weighted summation process, based on the system’s belief in how reliable the components are. This idea has also been extended to include situations where relying on a single signal rather than combining multiple signals may be advantageous^71^. Such an extended Bayesian framework shares certain similarities with our model, namely, the emphasis on the reliability of signals and the potential advantage of relying on a single signal. Also related to Bayesian models is the hybrid model of Harootonian et al.^32^, which has two components: A path integration system and a Kalman filter that independently estimate heading direction from idiothetic and allothetic information. Fitting the model to human behavior revealed that a hybrid strategy consistent with combining inputs when the mismatch is small and selecting one when the mismatch is large best described the behavior of all the participants.

While our model shares some similarities with the methods discussed above, it differs in several important respects. First, rather than assuming *a priori* knowledge about signal reliability, our model learns how to weight and use each signal through experience. Specifically, the degree to which each signal is relied upon emerges from the statistics of the task and environment, including the type and level of noise and whether signals are intermittent. Thus, how our model handles mismatches between signals is not fixed by design, but rather is a learned outcome: under high noise without intermittency, the network learns to rely predominantly on one signal while ignoring the other, while intermittency encourages the network to learn solutions that allow it to navigate flexibly under signal loss, encouraging integration even for larger mismatches. It is worth noting, nevertheless, that like any modeling framework, our RL model also comes with some implicit priors. Specifically, the choice of reward structure, network architecture, and training conditions all encode implicit priors that shape what the model learns, analogous to learning reliability parameters in Bayesian and Kalman-based approaches. Second is the question of how the hierarchy of vision over vector information can be explained. In probabilistic models, this dominance can be implemented by using a prior that biases the model towards vision^70^. By contrast, the hierarchy of vision in our model emerges through learning. We hypothesize that this hierarchy again arises because of the sensory encoding—although the goal vector is a more compact representation, precisely this property also makes it more likely to lead to more critical errors under noise. Finally, our model also goes beyond Bayesian models in that it operates in a closed loop, allowing us to study the interaction between sensory inputs, behavior, and internal representations in the network. Furthermore, although we simplify the encoding of the goal vector in the present study, it is in principle possible to extend the system to include more realistic implementations of the goal vector that can be learned, as we discuss above and partially demonstrate (Supplementary Fig. S1).

On the other hand, ring attractor models, which have been a predominant account of cue selection and integration, provide a complementary perspective at a more biologically plausible level of description^27,60,76,77^. These models are formulated at the implementation level, and can capture temporal dynamics—the activity bump can be updated by self-motion, persist when sensory input is absent, anchor to external cues, and the bump can naturally drift, which can explain accumulating errors in path integration. On the other hand, our model operates at a more abstract, algorithmic level and does not incorporate attractor dynamics, although an analogue may emerge in deep neural networks^78^. Contrastingly, rather than explaining how direction estimates are maintained moment-to-moment in a biologically plausible circuit, our model addresses how an agent learns from experience in a closed-loop to navigate under different noise types and intermittency conditions. A direct mapping between the sources of uncertainty in our model, i.e. pose and sensor noise, intermittency, and noise in the attractor models is not straightforward due to the different levels of description and underlying assumptions. Our model predicts that the pattern of integration for smaller discrepancies vs. competitive selection under conflict is not universally true and rather depends largely on the typical environmental and sensory uncertainty experienced. This, for instance, predicts strong inter-species differences in how cue conflicts are handled. How this property can be integrated into ring-attractor type models remains a direction for future investigation. Thus, ring attractors and our approach are better understood as complementary rather than competing accounts, addressing different aspects of the problem at different levels of analysis.

The operation in a closed loop is possible in our model due to the use of RL, which allows the agent to continuously learn and adapt its behavior based on feedback from the environment. However, this approach has its limitations; for example, learning occurs on very different time scales in RL models and in animals. The former often requires several thousands of trials to learn navigation tasks that would take mammals only a few training blocks. Moreover, the types of networks used for learning in deep RL algorithms are not entirely biologically plausible. RL also uses a specific optimality criterion, i.e. maximize cumulative reward, and previous modeling work has demonstrated that the optimal solution is constrained by the task and strategy available to the agent^41^. Regardless of these limitations, we believe that RL can be a useful modeling tool, especially for studying spatial navigation, because of some shared commonalities. For example, both the navigation problem and RL are goal-driven. In addition, they must also deal with balancing exploration and exploitation, i.e. repeating known routes, and must be able to deal with uncertainty. So, while specific features of RL may not match animal learning, we believe that it is a useful tool to understand the computational problem at hand and allows the exploration of different elements that contribute to navigation, such as the perception of sensory inputs, memory and replay, and decision-making. Moreover, RL models can be tuned and manipulated to explore various scenarios and test specific hypotheses. Making use of a neural network also enables the use of natural sensory representations (such as visual input) and the analysis of spatial representations that emerge in the network to support behavior. Together, these can provide valuable insights into underlying mechanisms and help uncover general principles governing different aspects of navigation.

Our model also has the potential to be integrated with existing models of path integration. In our simulations, the model has access to an already computed goal vector, which we assumed is the result of a separate computational process such as path integration. While path integration using self-motion is the best candidate for updating the goal vector, it could in practice be acquired from another process, for example, from path integration using optic flow, visual estimation, or an existing metric map of the environment. Animals could also use one of the above methods to occasionally re-calibrate and correct errors from path integration, or use some combination of the above. We partially addressed this issue by using two alternative versions of the model where the goal vector is learned through visual estimation (Supplementary Fig. S1). Another possible extension to have a more realistic implementation of the goal vector is to use a recurrent network that performs path integration, such as those used in Cueva & Wei^79^ and Banino et al.^35^, and use the position estimates of this network to compute the goal vector. Since these recurrent networks have been shown to develop grid-cell-like units, this extension could potentially also be used to study the interaction between grid and place cells in the model. This would also make fruitful the study of the dynamics of the interaction between the two signals in a more time-resolved manner along the navigation trajectory. We predict that the local interaction of signals in the dynamic case would qualitatively follow the averages we report in the current study.

In summary, a trained RL agent in our model can be seen as a high-level model of how an agent ought to combine different sources of information to achieve maximum performance in spatial navigation. Our simulation setup allows us to study the optimal strategies for information fusion in realistic settings while at the same time revealing emergent spatial representations that enable it to do so. The modeling results are qualitatively consistent with experimental results and frequently defy simple intuitions about the effects of removing a sensory input on neural spatial representations, and spatial learning and navigation.

## Methods and materials

### Task structure

Unless otherwise stated, all simulations used a simple navigation paradigm, called guidance, which is similar to a dry version of the Morris Water Maze. The task involved navigating to a fixed, unmarked goal location in the environment from different starting locations. At the start of each trial, a start location was assigned at random. To aid in localization, the walls of the enclosure were marked with distinct colors, which acted as distal landmarks. We used CoBeL-RL (Closed-loop simulator of complex behavior and learning based on reinforcement learning and deep neural networks)^80^ to generate the simulation environments and train the agents. The agent received visual input and goal vector information from the simulation environment at each time step. We defined two task schemes to evaluate the agent. In the first scheme, the visual or vector stream was randomly turned off or on every 30 trials, requiring the agent to learn to navigate using both streams simultaneously as well as separately. In the second scheme, both streams were available continuously, such that the agent could freely choose to select a single stream or combine them in order to navigate.

Training lasted for 4000 trials in total and each trial lasted 100 time steps, unless the trial was terminated earlier by correctly locating the goal. The agent received a +1 reward for reaching the goal node. The agent received a − 1 penalty (negative reward) for each step taken in order to motivate it to find shorter routes. If the agent selected an action that would have resulted in walking into a wall, it remained in place and received a − 1 penalty.

### Network model

The network model that was trained to solve the task is shown in Fig. 1. The network had two input layers that receive image and vector information, respectively. The two inputs were processed in separate streams before being combined. The visual stream processes visual information from the environment using a convolutional neural network (CNN)^81^. The CNN consisted of three layers with 32, 64, and 64 filters each, with kernel sizes of (5,5), (4,4), and (3,3), respectively. The vector stream received the goal vector through its input layer, which was connected to two fully connected feedforward network layers, which have 50 and 25 units, respectively. The two streams were then combined by a simple concatenation and further connected to a fully connected (FC) layer with 50 units and a dropout of 0.3, before an action selection layer with 12 units, which selected rotational and translational actions. A ReLU activation function^82^ was used throughout the network, except for the output layer, which used a linear activation function.

The actions available to the agent for interacting with the environment corresponded to the units of the action selection layer. Six rotational and six translational actions were available to the agent, which were aligned with the main axes of a hexagonal grid. Translational actions caused the agent to move to a neighboring node on the grid, while rotations caused the agent to change its heading direction to face a neighboring node.

### Visual and vector inputs

The visual stream received naturalistic RGB images that correspond to the agent’s current view of the environment. Each of the $48\times 12$-pixel images corresponded to a 240-degree field of view in the simulated environment. The vector stream received information about the goal vector. The input to this stream is $(d,\phi , \theta )$, where *d* is the distance to the goal, $\phi$ is the angle between the goal vector and a reference vector, and $\theta$ is the allocentric heading direction of the agent with the same reference vector.

The goal vector can also be learned. We demonstrated this using two alternative versions of the model (Supplementary Fig. S1). In the first version, the goal vector was learned from visual impressions of the environment by a neural network that was pre-trained using supervised learning. The network had the same convolutional neural network as the main model, followed by fully connected layers with 50, 25, and 12 units, respectively, and an output layer with 3 units corresponding to *d*, $\phi$, and $\theta$, which were used as input to the vector stream of the network depicted in Fig. 1. The second version used an identical network, except that the model was trained end-to-end using reinforcement learning (RL).

### Pose and sensor noise

To model pose noise, we introduced variability that directly perturbed the spatial signal provided to each input, proportional to the signal strength. The noise was sampled per step of the RL algorithm. For the vector stream, noise was added independently to each component of the goal vector as follows

$$\begin{aligned} d = d + \eta _d, \qquad \eta _d\sim N(0, \sigma ^{2}d^{2}) \end{aligned}$$

$$\begin{aligned} \phi = \phi + \eta _\phi , \qquad \eta _\phi \sim N(0, \sigma ^{2}\phi ^{2}) \end{aligned}$$

$$\begin{aligned} \theta = \theta + \eta _\theta , \qquad \eta _\theta \sim N(0,\sigma ^{2}\delta ^{2}) \end{aligned}$$

where $\delta$ is the angle between two consecutive heading directions on the grid, i.e. 60°.

For the visual stream, pose noise was introduced by perturbing the camera pose relative to the agent, such that the rendered camera image was sampled at

$$\begin{aligned} x_i = x_i + \eta _{x_{i}}, \qquad \eta _{x_{i}}\sim N(0, \sigma ^{2}x_i^{2}) \end{aligned}$$

for each component of the pose vector.

In contrast, sensor noise was designed to be independent of the position and instead introduced a general variability in the input signal. For the vector stream, sensor noise was injected at the first network layer by using a Gaussian Dropout layer (multiplicative, 1-centered Gaussian noise) inserted after the activation function. For the visual stream, sensor noise was applied independently to each image pixel by adding Gaussian noise scaled by the mean pixel value for each observation. The pixel values were subsequently normalized to remain within the range [0,255].

### Reinforcement learning

In RL, the model, referred to as an agent, interacts with its environment and receives feedback in the form of rewards or penalties based on its actions during training, and uses this to optimize the expected cumulative reward. This allows it to learn optimal behavior through trial-and-error—the agent learns from its own experiences and adapts its behavior accordingly. The RL algorithm used in our model is based on Q-learning^83^. In Q-learning, the agent learns to estimate Q-values, i.e., the value of each action $a_t$ in a given state $s_t$. These estimates are updated after each action using the following rule:

1 $$\begin{aligned} Q(s_t,a_t) \leftarrow Q(s_t,a_t) + \eta \left[ r_t + \gamma \max _{a}Q(s_{t+1},a) - Q(s_t,a_t) \right] \end{aligned}$$

We used the Deep Q-Network (DQN) algorithm^39^ to train the previously described network to navigate using visual and vector inputs. We used a learning rate $\eta = 0.001$ and a discount factor of $\gamma = 0.99$. The agent drew from an experience replay^84^ buffer of 3000 past experiences to learn from. A target network with soft updates ($\tau =0.01$) was used for stabilizing learning. In general, reinforcement learning agents must deal with what is known as the exploration-exploitation trade-off, which refers to the fact that agents must explore new actions to discover better strategies while also exploiting the actions that have yielded higher rewards in the past. We used a commonly employed policy, $\varepsilon$-greedy, with $\varepsilon = 0.3$ during training. In the test phase, the agent used a greedy policy, i.e., always picked the learned optimal action at every step.

### Behavioral analysis

We evaluated the behavior of the agent both during training, as well as after, in a test phase. A standard way to evaluate agent training performance is to examine the learning curves. In addition to this, in order to get a more fine-grained view of the agents’ performance, we defined a learning phase of 1000 trials in which learning converged in all models. A trial starts with the agent initialized at a random start location. The trial ends when the agent reaches the goal location, or after 100 timesteps without reaching the goal. We divided the first 1000 trials into five trial blocks with 200 trials each, and calculated the fraction of direct finding trials in each trial block. A direct finding trial is where the agent navigates from start to goal using the fewest possible number of actions, plus a tolerance of $30\%$ to allow for longer paths due to random exploration from the epsilon-greedy policy. Thus, a direct finding trial was defined as a trial where the path length $l\le 1.3 l_\text {min}$, where $l_\text {min}$ is determined by the lowest number of rotational and translational actions needed to move from the starting to the goal location on that trial. The performance of the agents was evaluated after training, in a test phase. The agent was tested in three different modes to assess which signals the agent had learned to use to solve the task. In the first mode, the agents received both the visual and vector signals, while in the other two modes, they received only one of the two signals for the entirety of the test phase. During testing, no noise was added to the visual and vector signals. The fraction of test trials in which the agent successfully navigated to the goal was used to measure performance.

### Statistical analyses

Statistical analyses were performed in Python3.14 using the packages scipy.stats and statsmodels. We report the mean ± SEM for all results.

Performance differences between agents from two different conditions were evaluated using two-sample *t*-tests. We applied a Bonferroni correction when comparing multiple conditions.

The effect of noise on learning in each condition was assessed using a one-way ANOVA across noise levels on the slope of the direct finding curves between the first and second trial-blocks as well as the area under the curve (AUC).

The relationship between noise levels and test performance was assessed using the Spearman’s rank correlation coefficient. We model the direct finding rate as a function of the proportion of place units using binomial regression, as the outcome is a bounded success rate.

For the modeling of lesions, we carried out a repeated-measures ANOVA, since the behavioral readouts were measured at multiple points during training. Furthermore, we adjusted for multiple comparisons using the Bonferroni method.

We use the following *p*-value legend throughout the text and in figures ***p<0.001, **p<0.01, *p<0.05.

### Simulation of cerebellar mice in light and dark conditions

We simulated the experiment described in Rochefort et al.^25^ using the Morris Water Maze task described above. If mice with cerebellar lesions have impaired path integration, we assume that this leads to a highly imprecise calculation of the goal vector. We simulated this by using agents with a highly noisy (variance = 0.8) vector signal. For the control mice, we used agents with a lower level of noise (variance = 0.1) in the vector stream. We do not use networks with zero noise since path integration is subject to accumulating errors and is thus inherently subject to some noise. We trained the networks for 1000 trials each and partitioned the learning phase into 5 learning blocks of 200 trials each. At the end of each learning block, the agents were tested under light and dark conditions. For the light condition, both the vector and visual stream were active, and for the dark condition only the vector stream was active. Each test phase lasted 100 trials, and the proportion of successful trials was computed.

### Simulation of rats with vestibular lesions

We hypothesized that lesions of the vestibular system lead to impaired path integration and simulated this using agents with the vector stream removed and compared their performance to control agents with low noise (variance= 0.1) in the vector stream. Two variations of the task were used, corresponding to the first and second criteria defined in the experiment by Stackman & Herber^42^. In the first variation, the agent always started from the same location, corresponding to a fixed holding box position in the experiment. In the second variation, four starting locations were used, again corresponding to the holding box positions. To enable comparison to the experimental results, we used an analogous performance measure. The criterion was defined as successful direct finding in three consecutive blocks of 16 trials, and the number of trials to meet the criterion during training was used to quantify the speed of learning.

### Analysis of spatial representations

We calculated spatial activity maps for each unit in the layer before the action selection layer by recording unit activation at each grid point on a $25 \times 25$ grid on the environment. As is common in experimental papers, the maps are smoothed using a Gaussian filter with a standard deviation of 2 pixels for visualization only. Raw maps before smoothing are shown in Fig. S3. This process is repeated for each of the six head directions, and the units are then classified as place cells, egocentric vector cells, head-direction modulated cells, and view-selective cells as outlined in Vijayabaskaran & Cheng^41^. The classification procedure comprises the following steps. First, we determined if the unit has a localized firing field for each of the head directions. This was done by first identifying the peak activity of the activation map and filtering out activity that was below 15% of the peak. If the resulting map had an region of activation that covered <50% of area of the arena and formed a contiguous region surrounding the peak, the map was considered to have a localized firing field. Next, if the location of peak activity remained constant over all head directions within 5% of the arena size, the unit was classified as place-like.

If the field center was always located at a fixed distance and angle relative to the agent’s head direction, the unit was classified as a vector unit, i.e. these units encode space in a. egocentric reference frame, a specific direction and distance from the agent, rather than an allocentric position in the environment.

If the unit was not vector-like, but the location of peak activity varied with the head direction, it was classified as a HD-Modulated unit. View-selective cells were defined as localized units that were active only during a single head direction or a restricted subset of continuous head directions. We also look for head direction cells, i.e. units that are tuned to a specific head direction irrespective of spatial location, but did not find any within our network. If a unit showed neither directional nor localized firing, it was classified as “other”.

## Supplementary Information

Supplementary Information.

## Data Availability

All simulations were carried out using the CoBeL-RL framework available here: https://github.com/sencheng/CoBeL-RL Code for the simulations is available here: https://github.com/sandhiyavb/sensory-integration (https://doi.org/10.5281/zenodo.21364468)
