# Supplementary material for: A deep reinforcement learning account of competition and integration of visual and goal vector signals for spatial navigation
Source: Sci Rep. 2026 Jul 27;16:23351. doi: 10.1038/s41598-026-63080-3 (PMC13408606; doi:10.1038/s41598-026-63080-3)
Supplement: Supplementary file 1 — Supplementary Information. [file 41598_2026_63080_MOESM1_ESM.pdf]

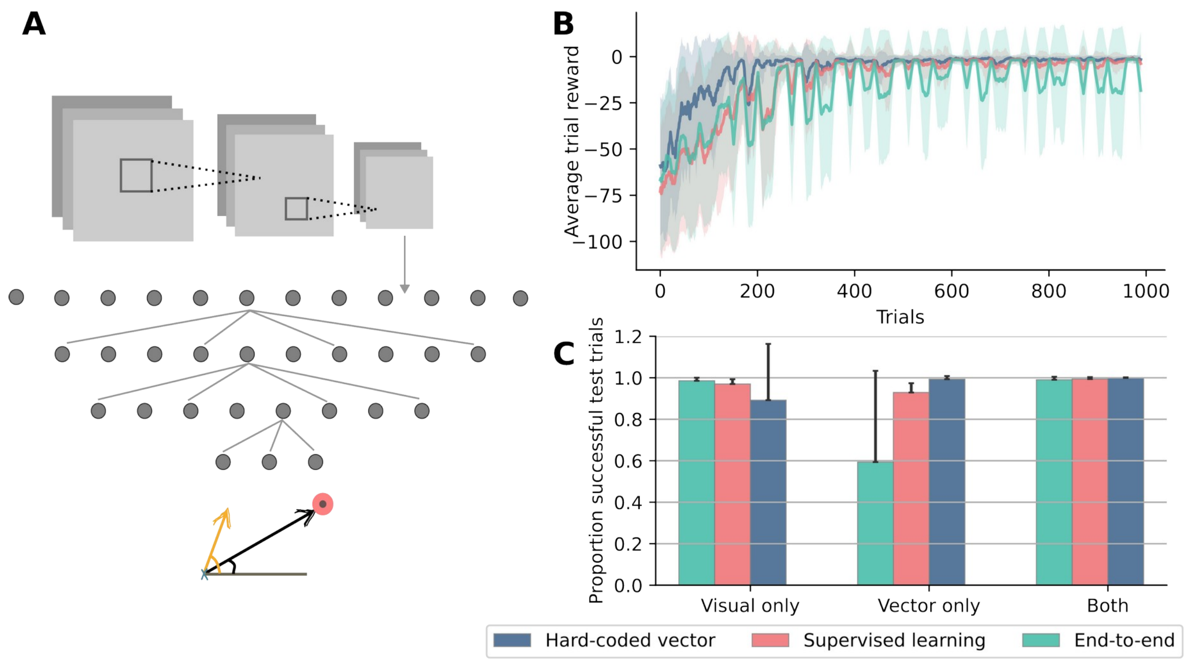

Figure S1: The effect of the vector encoding scheme on learning and performance. A: The network used to learn a vector representation. The network is either trained end-to-end as part of the vector stream in the full model using reinforcement learning, or it is pre-trained to predict the goal vector using supervised learning. **B:** Learning curves using the three vector encoding schemes. The network successfully learns the task using all three schemes, however, learning is slower and noisier for the end-to-end scheme and is fastest while using a hard-coded vector.

C: Test performance of the three vector encoding schemes measured with both inputs, and using either one of the inputs only. While the performance for all three schemes is comparable when both inputs are available, the reliability of the vector encoding scheme determines how well the network performs when only vector information is available. The better the vector encoding, the worse the network performs when only the visual stream is active.

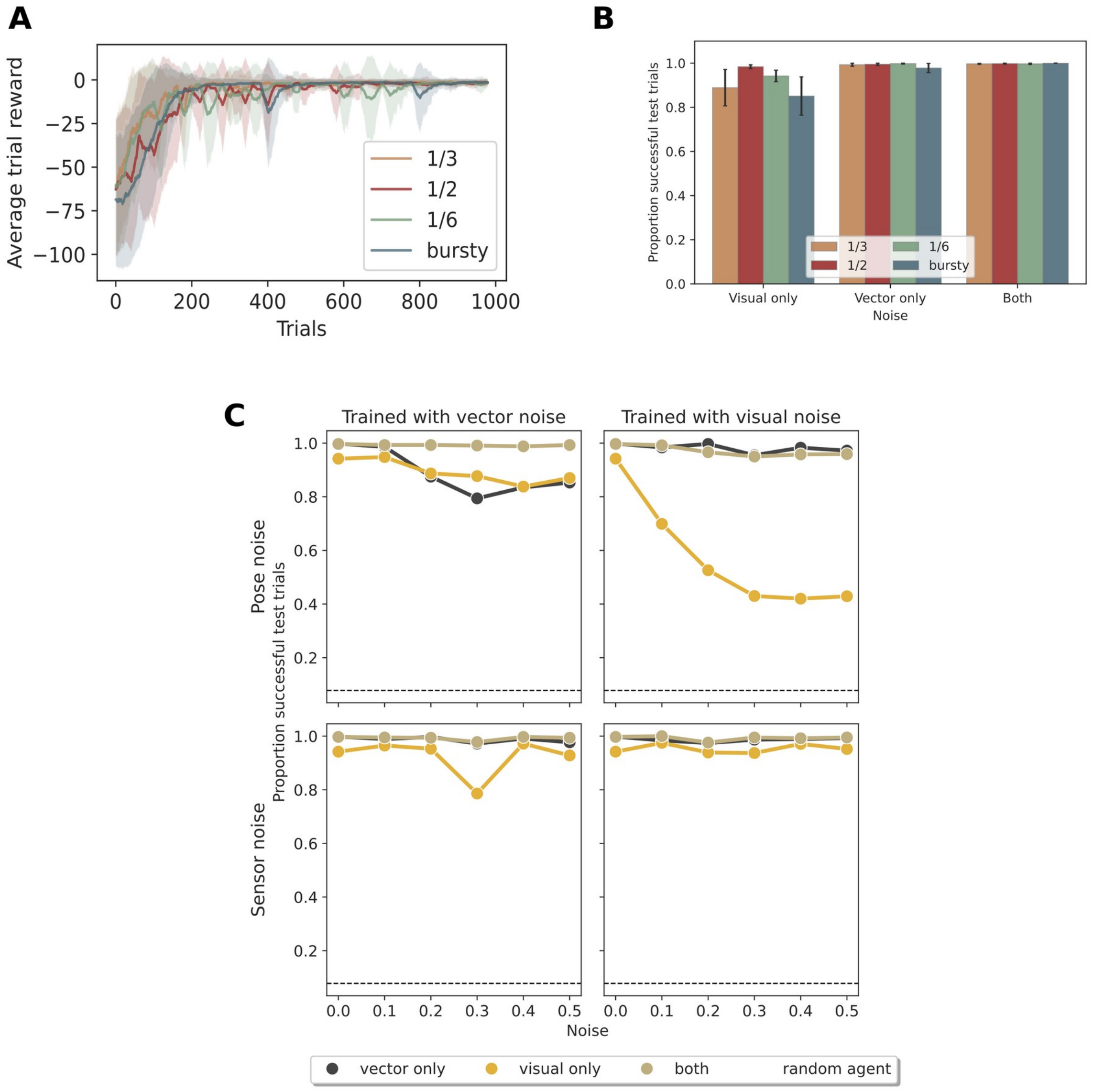

Figure S2: Effect of different intermittency schedules on performance A: Learning curves for four different intermittency schedules. Fraction indicates fraction of time signal loss is experienced. **B:** Test performance when each signal is removed. Bars indicate mean, error bars indicate SEM. **C:** Effect of noise on performance for the 1/6 intermittency schedule.

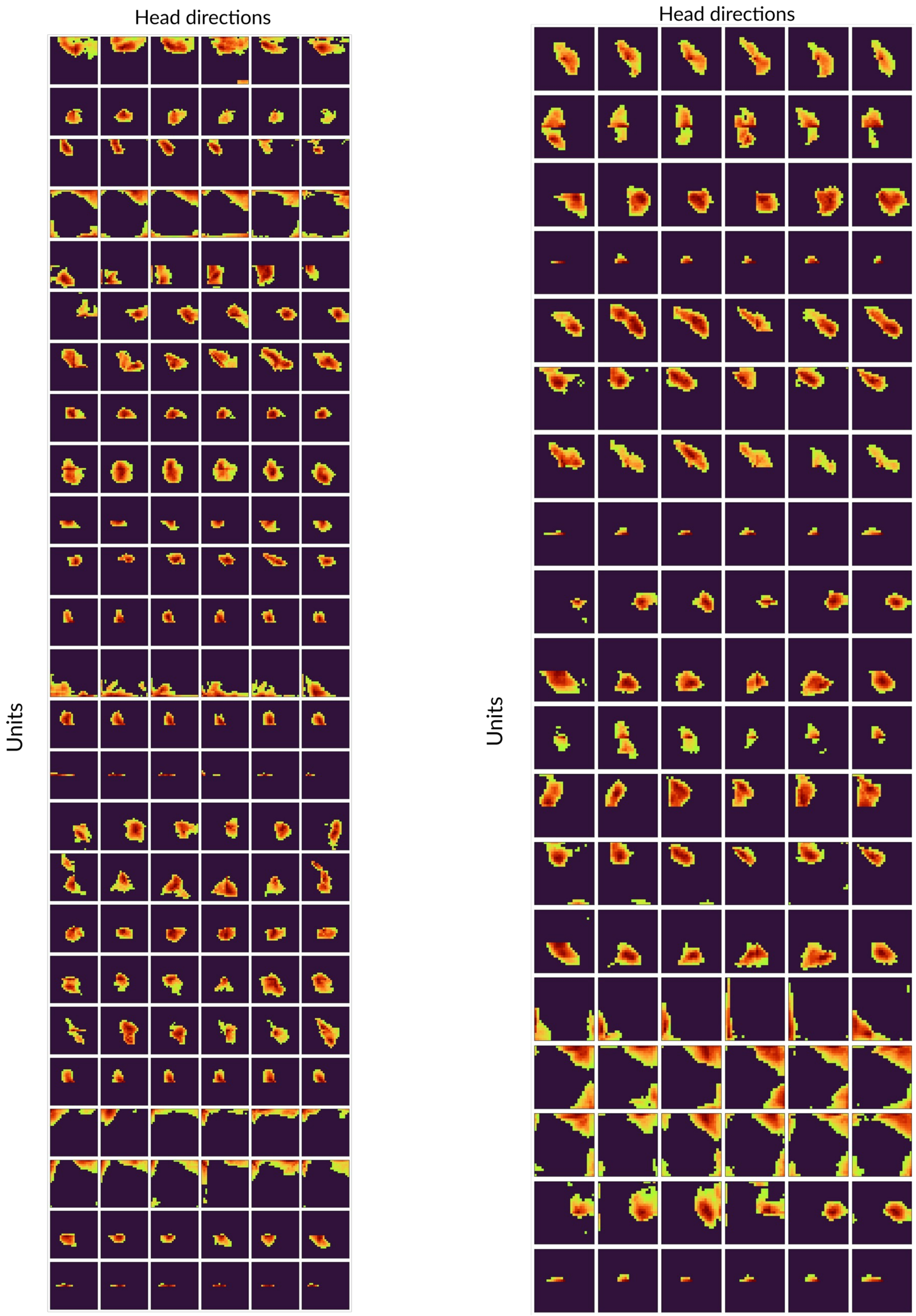

Figure S3: Raw place cell maps. Place cell maps before smoothing is applied from an example run for the intermittent (left) and continuous (right) navigation tasks.

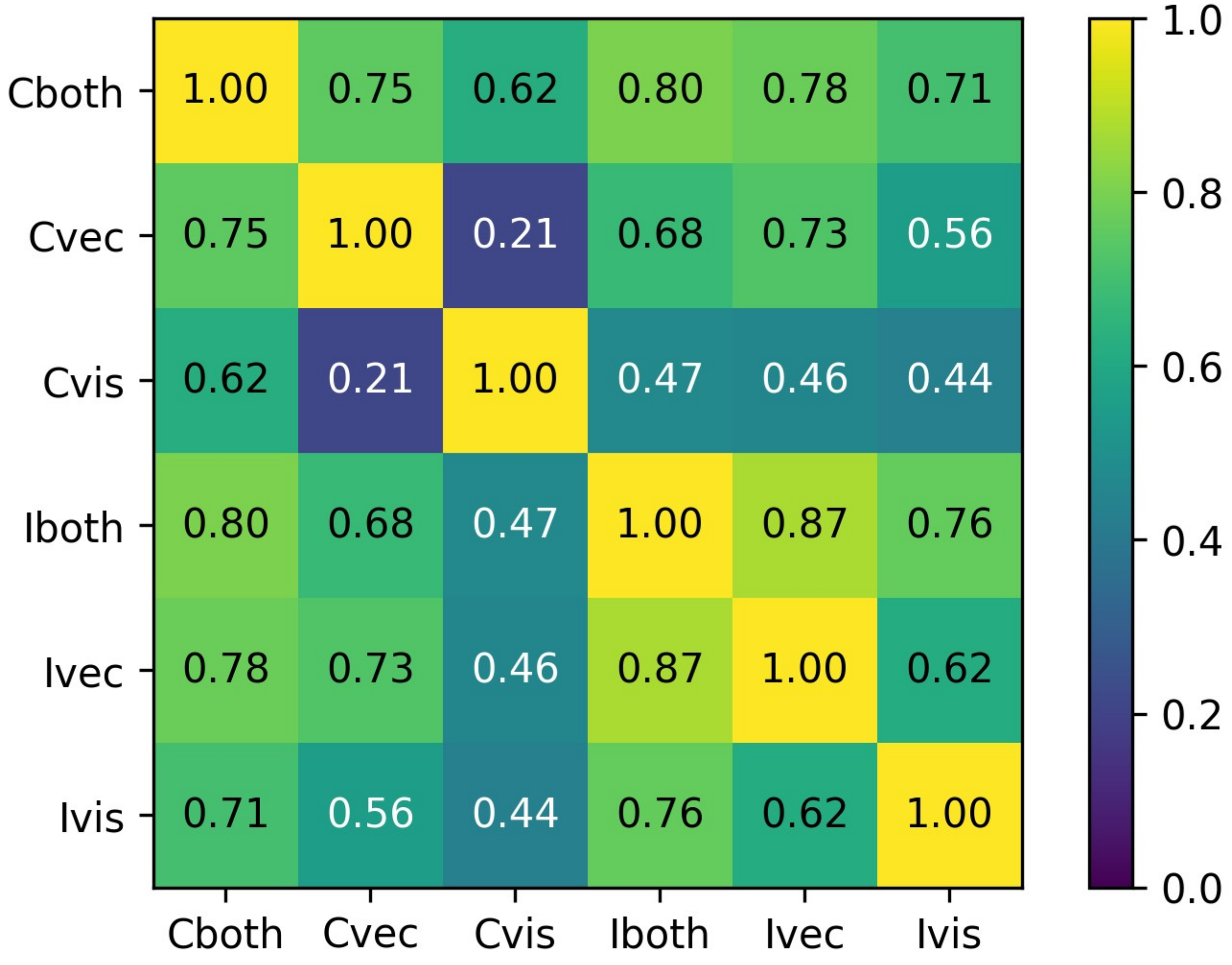

Figure S4: Average similarity between models: Similarity matrix showing the average similarity score (linear CKA) across models between representations in the penultimate layer in the continuous (C) and intermittent (I) navigation tasks, when both, vector only (vec) and visual only (vis) are present.
